# Effect of Chitosan Modification and Support Type on the Catalytic Properties of Supported Palladium Catalysts in Hydrogenation of 2-Propen-1-ol

**DOI:** 10.3390/molecules31122028

**Published:** 2026-06-10

**Authors:** Akzhol Naizabayev, Eldar Talgatov, Assemgul Auyezkhanova, Arlan Abilmagzhanov, Sandugash Akhmetova, Alima Kenzheyeva, Raiymbek Yersaiyn

**Affiliations:** Dmitry Vladimirovich Sokolsky Institute of Fuel, Catalysis and Electrochemistry, Kunaev Str. 142, Almaty 050010, Kazakhstan; a.naizabayev@ifce.kz (A.N.); e.talgatov@ifce.kz (E.T.);

**Keywords:** supported palladium catalysts, chitosan, hydrogenation, 2-propen-1-ol, propanol, propionic aldehyde

## Abstract

Palladium catalysts modified with chitosan (CS) and supported on MgO, SiO_2_, TiO_2_, and Al_2_O_3_ were prepared by a precipitation method and evaluated in the low-temperature hydrogenation of 2-propen-1-ol. Chitosan was first deposited onto the oxide supports by adjusting the suspension pH to 7.5, followed by immobilization of palladium via reductive deposition using NaBH_4_. For comparison, analogous non-modified catalysts were synthesized. Physicochemical characterization (TGA, XPS, HAADF-STEM, SEM, viscosimetry, and elemental analysis) confirmed successful incorporation of Pd (1 wt.%) and CS (10 wt.%). HAADF-STEM revealed that Pd particle size and aggregation strongly depended on the support nature, with the most uniform distribution observed for Al_2_O_3_-supported catalysts. Chitosan modification reduced Pd nanoparticle size from 4–11 to 3–4 nm and improved dispersion. XPS showed a pronounced increase in the fraction of oxidized Pd species for the Al_2_O_3_- and TiO_2_-supported catalysts, whereas only minor changes were observed for the SiO_2_-based system. For unmodified catalysts, the nature of the oxide support strongly influenced their performance, resulting in a wide variation in catalytic activity (TOF = 1650–13,100 h^−1^) and selectivity toward propanol (65–75%). Chitosan modification resulted in a support-dependent convergence of catalytic activity (TOF = 3130–8840 h^−1^) and selectivity (76–81%). Stability tests were performed for Pd–CS(10%)/MgO and Pd–CS(10%)/Al_2_O_3_, which showed stable performance over 20 cycles without significant loss in catalytic activity. Overall, chitosan modification significantly influences Pd dispersion, oxidation state, and catalytic performance, with effects strongly dependent on the oxide support.

## 1. Introduction

Hydrogenation of unsaturated compounds plays a key role in the modern chemical industry, serving as a foundation for the production of a wide range of products in the pharmaceutical, chemical, and polymer sectors [1,2,3,4,5]. In the context of sustainable development and the transition to renewable raw materials, the catalytic upgrading of biomass-derived compounds, particularly C_3_ platform molecules, is of significant interest [5,6,7,8]. Allyl alcohol can be obtained by selective hydrogenation of acrolein, which is formed during the dehydration of glycerol, a large-scale by-product of biodiesel fuel production [5,8,9,10,11]. The transformation of allyl alcohol into 1-propanol enables the stabilization of highly reactive unsaturated oxygenates into a more valuable and industrially relevant alcohol [6,11]. Therefore, this reaction is highly relevant to the catalytic upgrading strategies in biomass valorization. In addition, allyl alcohol is widely used as a precursor for the synthesis of various valuable chemical compounds. Its hydrogenation product, 1-propanol, is a versatile solvent and an important chemical intermediate used across multiple industries [12]. Owing to the broad range of applications of both compounds, the hydrogenation of allyl alcohol is of considerable scientific and industrial interest.

The hydrogenation of allyl alcohol (2-propen-1-ol) can proceed via several competitive reaction pathways. In addition to the direct hydrogenation of the C=C double bond to form 1-propanol, metal-catalyzed isomerization of allyl alcohol to propanal is possible, carried out by intramolecular hydrogen transfer. The resulting propanal, in turn, can be further hydrogenated to 1-propanol by reduction of the C=O carbonyl group [12,13,14,15,16]. Such an interplay between C=C hydrogenation, C=O hydrogenation, and isomerization reactions is often observed during the hydrogenation of biomass-derived oxygenates [7]. In this regard, allyl alcohol represents a convenient model compound for evaluating hydrogenation catalytic systems in terms of activity, selectivity, and the ability to direct the reaction along a desired pathway. The study of this reaction contributes to a deeper understanding of the transformation mechanisms of unsaturated oxygenates and to the development of effective catalytic upgrading strategies for biomass processing [7,17,18]. It should be noted that there are relatively few recent studies devoted to the investigation of the hydrogenation of 2-propen-1-ol. In the studies [6,12,15,19,20,21,22,23], palladium catalysts were employed for the hydrogenation of allyl alcohol. A correlation between the structure of surface ligands and catalytic selectivity was demonstrated. In addition, the effects of ligand structure and conformation, solvent nature, and the introduction of additives on the activity and selectivity of catalysts based on Pd nanoparticles were examined.

In our previous studies, we demonstrated that polymer-modified Pd/ZnO, Pd/MgO, and Pd/SBA-15 catalysts exhibit promising performance in allyl alcohol hydrogenation [14,24]. The fixation of polymer-metal complexes on the surfaces of the solid supports has gained substantial interest as a promising approach for designing heterogeneous catalysts with incorporated metal nanoparticles. The primary role of the polymer in the synthesis of metal nanoparticles on modified supports is to ensure high metal dispersion and to stabilize the nanoparticles against aggregation [14,24,25,26].

Recent studies highlight the potential of chitosan in the design of heterogeneous catalysts. Chitosan-based catalysts have attracted significant interest due to the distinctive properties of chitosan (CS), such as its chelating ability, non-toxicity, renewability, and high availability [27,28,29,30,31]. In addition, the unique chemical structure and functional properties of chitosan enable the preparation of catalytic systems via different synthetic approaches, thereby broadening its application in catalysis. The presence of reactive amino and hydroxyl groups allows its chemical modification (functionalization and cross-linking), while its pH-dependent solubility and phase behavior in aqueous media provide additional flexibility in catalyst design. Taken together, these properties enable the use of chitosan either as a standalone polymeric support or as a surface modifier of inorganic oxides. In metal–polymer/support systems, chitosan can be deposited onto supports in a controlled manner, forming hybrid catalytic materials with a well-defined polymer content [31].

In our previous study [24], chitosan in palladium catalytic systems was primarily considered as a modifier, stabilizer of the active phase, and complexing agent. It was also demonstrated that the catalytic activity of such systems strongly depends on the nature of the oxide support. However, the chitosan content in those systems was comparable to that of palladium (approximately 1 wt.%), which made it difficult to clearly distinguish the contributions of the polymer modifier and the support effect to the overall catalytic performance.

In the present work, the chitosan content was intentionally increased, and oxide supports of different nature (MgO, SiO_2_, TiO_2_, and Al_2_O_3_) were employed. This approach enabled a more detailed elucidation of the individual roles of polymer modification and oxide support in governing the structure–activity relationships of Pd-based catalysts, revealing their distinct and support-dependent effects on catalytic activity and selectivity.

## 2. Results and Discussion

### 2.1. Synthesis of Palladium Catalysts Deposited on Support Materials and Their Characteristics

The palladium catalysts deposited on support materials were developed. As a support, both commercial oxides (MgO, SiO_2_, anatase TiO_2_) and synthesized alumina (Al_2_O_3_) sample were used. The synthesized alumina was prepared by precipitation in ethanol according to the method described in ref. [32].

**Figure 1:**
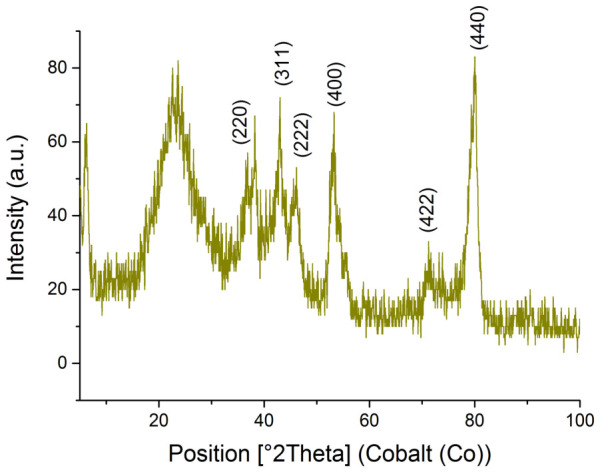
Diffractogram of the synthesized Al_2_O_3_.

Figure 1 shows the X-ray diffraction (XRD) pattern of the synthesized alumina, the characteristic diffraction peaks observed at 38.2°, 43.0°, 46.0°, 53.2°, 71.2°, and 79.9° correspond to the (220), (311), (222), (400), (422), and (440) crystallographic planes of cubic γ-Al_2_O_3_ (JCPDS card No. 79-1558) [32,33]. The noticeable broadening of these peaks indicates the nanoscale nature of the obtained particles [34]. In addition, a broad diffuse reflection in the 20–30° 2θ range is observed, which can be attributed to the presence of an amorphous phase composed of very small alumina particles with low crystallinity or nanocrystalline domains. The average crystallite size, estimated using the Scherrer equation based on the full width at half maximum (FWHM) of the most intense (440) reflection, was found to be approximately 23 nm.

The palladium catalysts were prepared by deposition of chitosan (CS) and palladium ions onto the surface of metal oxides (MgO, SiO_2_, TiO_2_, and synthesized Al_2_O_3_). The deposition of Pd ions was carried out using sodium borohydride as a reducing precipitant. Unmodified Pd/MgO, Pd/TiO_2_, Pd/Al_2_O_3_, and Pd/SiO_2_ catalysts were synthesized using the same procedure, except that no polymer was introduced. According to EDX elemental analysis the complete fixation of palladium on the surface of supports was observed (Table 1). The palladium content in the resulting catalysts was consistent with the calculated values and amounted to approximately 1 wt.%.

**Figure 2:**
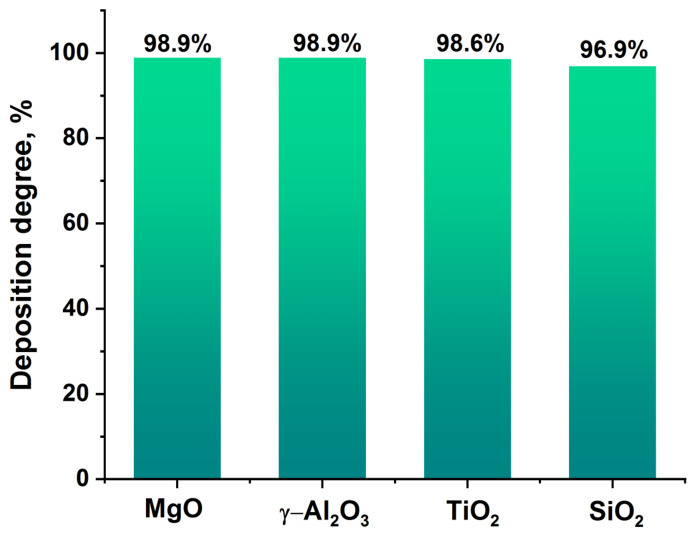
Deposition of chitosan on supports materials.

Chitosan deposition onto the supports (MgO, Al_2_O_3_, SiO_2_, and TiO_2_) was carried out by a precipitation method with adjustment of the solution pH to 7.5. The amounts of chitosan introduced was taken from the calculation to obtain the catalysts with the polymer content of 10 wt.%. Prior to alkalization, the upper part of the suspension was viscous and turbid. Suspended support particles accumulated in the upper layer of the suspension were also observed. After adjusting the pH to 7.5, a swollen curd-like loose precipitate was formed, while the upper liquid phase became visually transparent, indicating nearly complete deposition of chitosan onto the supports. The amount of deposited chitosan was estimated from the viscosity of the mother liquor using a calibration curve. All viscosity measurements were carried out at a constant temperature of 20 °C. According to the obtained results, the viscosity of the remaining mother liquor was close to that of pure water, confirming that only negligible amounts of dissolved chitosan remained in solution after precipitation. The degree of chitosan deposition on the oxide surfaces ranged from 96.9 to 98.9% (Figure 2).

**Figure 3:**
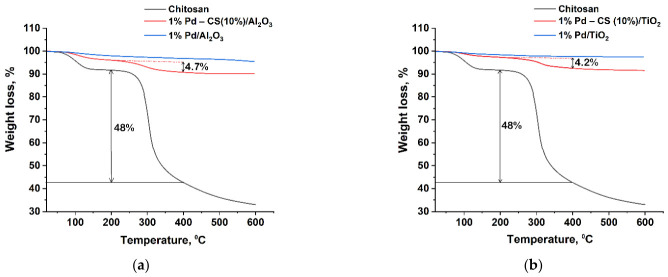

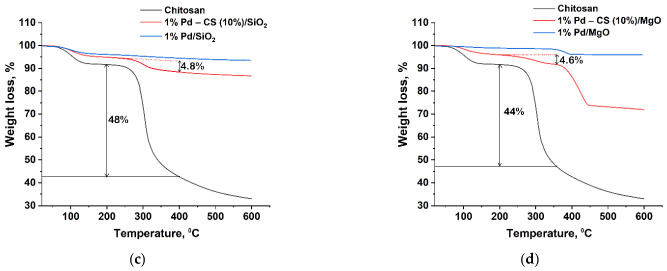
TGA of the chitosan and palladium catalysts: chitosan, 1%Pd–CS(10%)/Al_2_O_3_, 1%Pd/Al_2_O_3_ (**a**); chitosan, 1%Pd–CS(10%)/TiO_2_, 1%Pd/TiO_2_ (**b**); chitosan, 1%Pd–CS(10%)/SiO_2_, 1%Pd/SiO_2_ (**c**); chitosan, 1%Pd–CS(10%)/MgO, 1%Pd/MgO (**d**).

The presence of polymer in the obtained composites was confirmed by thermogravimetric analysis (TGA) (Figure 3). The results showed that upon heating to 200 °C, all samples lose approximately 2–5% of their mass due to the removal of corresponding to the removal of physically adsorbed moisture and residual solvent species trapped in the polymer matrix and on the catalyst surface. In this temperature range, the polymer-free samples exhibited lower weight loss (1–3%) compared to their polymer-modified counterparts (4–5%). For the chitosan-modified samples, further temperature increase leads to the onset of thermal decomposition of the polymer. In the range of 200–400 °C the 1%Pd–CS(10%)/Al_2_O_3_, 1%Pd–CS(10%)/TiO_2_, and 1%Pd–CS(10%)/SiO_2_ catalysts lose 4.7%, 4.2% and 4.8%, respectively of the mass (Figure 3a–c). Considering that pure chitosan loses approximately 50% of its mass within this temperature range, the polysaccharide content in these catalysts can be estimated to be 9–11%. This value is close to the nominal one (10%), indicating efficient modification of the catalysts with the polymer. The thermal behavior of the MgO supported sample differs slightly from those of the Al_2_O_3_-, TiO_2_- and SiO_2_-based catalysts. For the 1%Pd–CS(10%)/MgO catalyst, three main weight-loss stages were observed (Figure 3d). The first stage in the range of 20–200 °C was attributed to the removal of physically adsorbed water. The second stage between 200 and 360 °C corresponded mainly to the thermal decomposition of chitosan. A third pronounced weight-loss region above 360 °C and extending up to 500 °C was associated both with further decomposition of the carbonized chitosan residue and with transformations in the inorganic phase, including dehydroxylation of Mg(OH)_2_ and decomposition of MgCO_3_ accompanied by CO_2_ evolution [35]. Therefore, the chitosan content in this catalyst was estimated using the 200–360 °C temperature interval. In this range, the catalyst exhibited a weight loss of 4.6%, which corresponds to approximately 10 wt.% chitosan, considering that pure chitosan loses about 44% of its mass within the same temperature interval.

The prepared catalysts were also analyzed for carbon, nitrogen, and hydrogen contents using elemental organic analysis (Table 2). For comparison, pure chitosan was also analyzed. The elemental composition of chitosan was found to be 42.1 wt.% for carbon, 7.5 wt.% for hydrogen, and 7.4 wt.% for nitrogen, which is in good agreement with literature data [36]. Assuming a nominal chitosan loading of 10 wt.% in the catalysts, the expected elemental contents in the final materials would be approximately 4.2 wt.% C, 0.75 wt.% H, and 0.74 wt.% N. The detection of nitrogen (0.2–0.6 wt.%) in all catalyst samples confirms successful incorporation of chitosan, since the pristine supports do not contain nitrogen. Although the experimentally determined nitrogen contents are lower than the theoretical value, the carbon contents (4.5–5.6 wt.%) are close to the calculated values for a 10 wt.% chitosan loading. The somewhat higher hydrogen contents (0.8–1.6 wt.%) may be associated with adsorbed water and surface hydroxyl groups on the oxide supports.

A recalculation based on the carbon content indicates that the actual chitosan loading in the catalysts is in the range of 10–13 wt.%. This suggests efficient immobilization of the polymer on the support surface. The obtained values are in good agreement with the results of viscometry and TGA, further supporting the successful and stable deposition of chitosan on the metal oxide supports.

**Figure 4:**
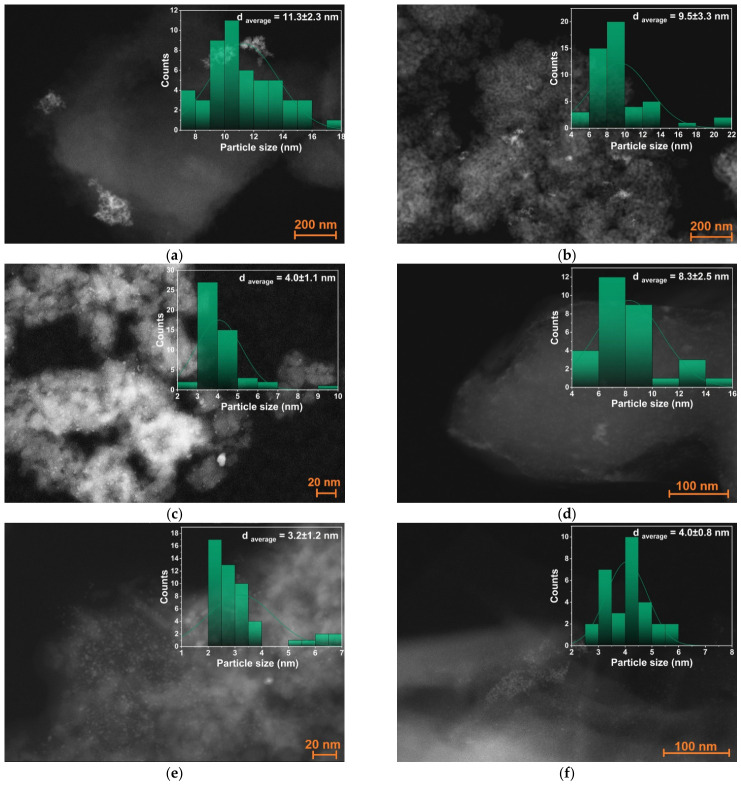
HAADF-STEM images and corresponding Pd particle size distribution histograms (insert) of 1%Pd-SiO_2_ (**a**), 1%Pd/TiO_2_ (**b**), 1%Pd/Al_2_O_3_ (**c**), 1%Pd/MgO (**d**), 1%Pd-CS(10%)/Al_2_O_3_ (**e**) and 1%Pd-CS(10%)/MgO (**f**).

Figure 4a–f presents HAADF-STEM micrographs and the corresponding Pd particle size distribution histograms of 1%Pd/SiO_2_, 1%Pd/TiO_2_, 1%Pd/Al_2_O_3_, 1%Pd/MgO, 1%Pd-CS(10%)/MgO and 1%Pd-CS(10%)/Al_2_O_3_ catalysts. The 1%Pd/SiO_2_ catalyst is characterized by the formation of large Pd-containing aggregates with sizes of approximately 100–200 nm (Figure 4a). These aggregates consist of smaller palladium nanoparticles with an average size of about 11 nm, while isolated Pd particles are rarely observed. In the case of 1%Pd/TiO_2_, smaller and less compact aggregates with sizes of about 30–40 nm are observed (Figure 4b). These aggregates are composed of Pd nanoparticles with an average size of approximately 9 nm. In contrast to SiO_2_, individual particles and small clusters are also detected on the TiO_2_ surface. A markedly different morphology is observed for the 1%Pd/Al_2_O_3_ catalyst (Figure 4c), where Pd nanoparticles with an average size of about 4 nm are uniformly distributed over the alumina surface without pronounced aggregation. The particles exhibit a nearly spherical morphology and relatively narrow size distribution, indicating efficient stabilization of Pd species on the alumina support. The 1%Pd/MgO catalyst (Figure 4d) contains Pd nanoparticles with an average size of approximately 8.3 nm distributed over the MgO surface. At the same time, the formation of larger aggregates ranging from 10 to 30 nm is observed in different regions of the support, indicating partial agglomeration of Pd species. The observed variation in Pd particle size and aggregation behavior across different supports can be rationalized by a combination of electrostatic surface properties, metal–support interactions, and textural characteristics. Among the investigated oxide supports, Al_2_O_3_ exhibits the highest Pd dispersion despite its moderate specific surface area (~94 m^2^/g). This behavior can be attributed to the relatively high point of zero charge (PZC ≈ 6.8) and the presence of tetrahedrally coordinated Lewis acidic Al^3+^ surface sites capable of stabilizing Pd species through Pd–O–Al interactions [37,38]. Such interactions may suppress the migration and growth of Pd species and inhibit nanoparticle aggregation during reduction. In contrast, TiO_2_ possesses a similar specific surface area (~88 m^2^/g) but exhibits noticeably lower Pd dispersion. This difference suggests that specific surface area alone is not the dominant parameter governing Pd nanoparticle stabilization. Compared with alumina, TiO_2_ has a lower point of zero charge (PZC ≈ 4.9) [37], resulting in weaker adsorption of anionic [PdCl_4_]^2−^ precursor species [39]. Consequently, Pd nucleation may partially occur away from the support surface, promoting particle growth and aggregation before deposition onto the oxide surface. An even stronger aggregation tendency is observed for SiO_2_, despite its very high specific surface area (~517 m^2^/g). The low PZC value of silica (PZC ≈ 3.2) indicates a strongly acidic surface [36] with limited adsorption affinity toward anionic [PdCl_4_]^2−^ complexes. As a result, stabilization of Pd precursor species on the support surface becomes inefficient, favoring uncontrolled particle growth and aggregation during reduction. These observations indicate that the electrostatic properties of the support surface and precursor adsorption behavior may play a more important role in Pd dispersion than the specific surface area itself. The behavior of MgO reflects the opposite situation, where favorable surface chemistry is combined with severe textural limitations. Its strongly basic surface (PZC ≈ 10–12) [40], together with the reported ability of Pd species to induce and occupy oxygen vacancies on MgO [41], is expected to favor the stabilization of Pd nanoparticles. However, the very low specific surface area of MgO (~10 m^2^/g) results in a high local concentration of Pd precursor species, facilitating particle growth and aggregation during reduction. Thus, although electrostatic interactions and basic surface sites may promote Pd stabilization, the limited available surface area restricts efficient spatial separation of Pd nanoparticles.

The introduction of chitosan modifies the morphology of Pd species on oxide supports. For the 1%Pd-CS(10%)/Al_2_O_3_ catalyst (Figure 4e), the average Pd particle size decreases only slightly from ~4 to ~3 nm, while the overall particle distribution remains relatively uniform and comparable to that observed for the polymer-free alumina-supported sample. However, the Pd-containing species in the chitosan-modified sample appear less contrast-intensive in the HAADF-STEM images, which may indicate partial coverage or embedding of Pd nanoparticles within the low-contrast polymer matrix. A more pronounced effect of chitosan is observed for the MgO-supported catalyst. In the 1%Pd-CS(10%)/MgO sample (Figure 4f), the average Pd particle size decreases from ~8 to ~4 nm, while large aggregates are no longer observed. The Pd nanoparticles appear distributed within a semi-transparent low-contrast matrix, presumably associated with a chitosan-derived polymer layer formed on the oxide surface. This morphology suggests that the polymer phase contributes to the stabilization and spatial separation of Pd nanoparticles. Such behavior may be attributed to the ability of the amino and hydroxyl functional groups of chitosan to interact with Pd precursor species, thereby contributing to the stabilization of Pd nanoparticles and limiting their growth during reduction. In particular, the polymer matrix may partially suppress Pd aggregation and Ostwald ripening by restricting nanoparticle migration within the hydrogen-bonded chitosan network. The formation of Pd nanoparticles with sizes of ~3–4 nm is consistent with previously reported Pd/chitosan systems [42].

**Figure 5:**
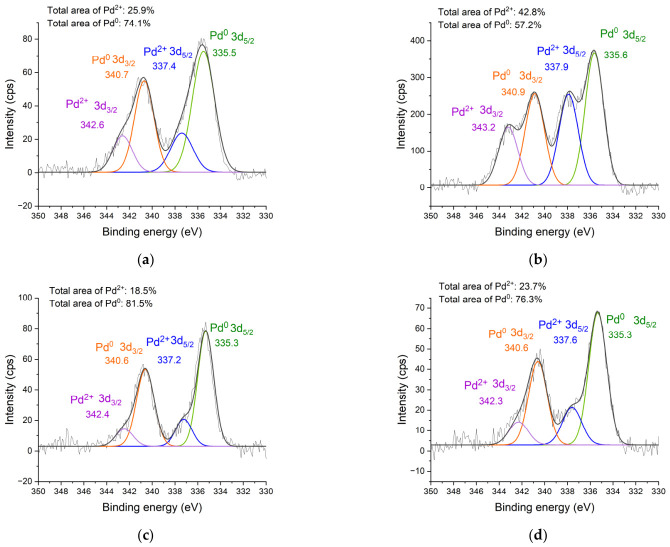

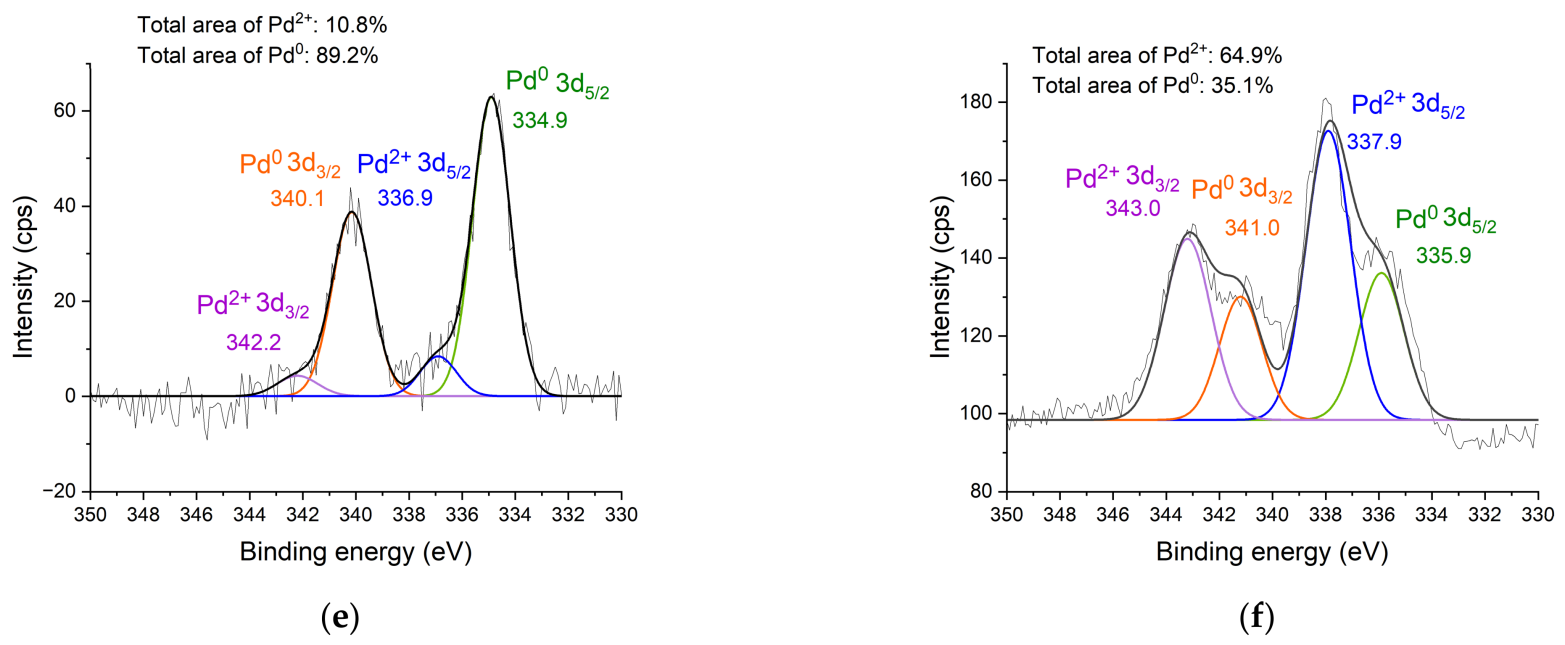
XPS spectra of Pd*3d* of the 1%Pd/Al_2_O_3_ (**a**), 1%Pd-CS(10%)/Al_2_O_3_ (**b**), 1%Pd/SiO_2_ (**c**), 1%Pd–CS(10%)/SiO_2_ (**d**), 1%Pd/TiO_2_ (**e**) and 1%Pd-CS(10%)/TiO_2_ (**f**).

In order to assess the state of palladium during the hydrogenation process, the 1%Pd/Al_2_O_3_, 1%Pd/TiO_2_, 1%Pd/SiO_2_, 1%Pd-CS(10%)/Al_2_O_3_, 1%Pd-CS(10%)/TiO_2_, Pd-CS(10%)/SiO_2_ catalysts were studied using X-ray photoelectron spectroscopy (XPS). XPS analysis of 1%Pd-CS(10%)/MgO and 1%Pd/MgO catalysts was complicated by the overlap of Pd 3d signals with Mg KLL Auger transitions, which made interpretation difficult [43,44]. XPS data illustrate the different oxidation states of Pd existing on the surface of the catalysts (Figure 5).

The Pd 3d spectra of all investigated catalysts exhibit characteristic spin–orbit doublets with an energy splitting of ~5.2–5.3 eV. The peaks located at ~334.9–335.9 and ~340.1–341.0 eV are assigned to Pd^0^ 3d5/2 and Pd^0^ 3d3/2 components, respectively, whereas the peaks at ~336.9–337.9 and ~342.2–343.2 eV correspond to Pd^2+^ 3d5/2 and Pd^2+^ 3d3/2 species, respectively (Figure 5). For all investigated polymer-free catalysts, the Pd 3d spectra are dominated by metallic Pd species (Figure 5a,c,e). However, minor contributions of oxidized Pd species are also observed despite the reduction treatment with sodium borohydride. This observation is consistent with a previous report for Pd/Al_2_O_3_, Pd/SiO_2_, and Pd/TiO_2_ systems [37], where the presence of oxidized Pd species was associated with metal–support interactions and partial stabilization of interfacial Pd species through interaction with oxygen species at the oxide surface. In addition, the presence of oxidized Pd species may also be partially attributed to re-oxidation of palladium during storage under ambient air conditions, as previously reported for commercial Pd/Al_2_O_3_ catalysts [45]. The Pd^2+^ fractions for Pd/Al_2_O_3_ and Pd/SiO_2_ are comparable, whereas Pd/TiO_2_ exhibits a lower Pd^2+^ content together with a slight negative shift in Pd 3d binding energy relative to the other supports. Similar negative binding energy shifts in Pd/TiO_2_ systems have previously been attributed to electron donation from TiO_2_ to Pd nanoparticles and strong metal–support interactions [46].

Modification with chitosan led to an increase in the fraction of oxidized Pd species for all investigated supports, although the magnitude of this effect was found to depend on the nature of the oxide support (Figure 5b,d,f). The most pronounced increase was observed for the TiO_2_-supported catalyst, where the Pd^2+^ contribution increased from 10.8% to 64.9%. At the same time, the negative binding energy shift observed for the polymer-free TiO_2_-supported catalyst disappeared after chitosan modification, which may be attributed to coordination interactions between Pd species and the amino and hydroxyl functional groups of chitosan [47,48] (Figure 5e,f). For the 1%Pd-CS(10%)/Al_2_O_3_ catalyst (Figure 5b), the Pd^2+^ fraction also increased significantly compared with the polymer-free analogue. Such behavior is consistent with the HAADF-STEM results, which indicate modification of the local environment of Pd species after chitosan incorporation (Figure 4c,e). The effect of chitosan modification was less pronounced for the SiO_2_-supported catalyst, which may be related to the textural properties of the oxide supports. In particular, Al_2_O_3_ and TiO_2_ exhibit comparable specific surface areas (88–94 m^2^/g), whereas SiO_2_ possesses a significantly higher specific surface area (517 m^2^/g). Nevertheless, a moderate change in the oxidized Pd fraction was still observed for this catalyst (Figure 5c,d). These observations are supported by a recent fundamental study, where it was shown that Pd on nitride supports exhibits a higher oxidation state than on oxide supports. In that study, this behavior was attributed to the existence of Pd in distinct local coordination environments (Pd−N_x_ on nitride and Pd−(OH)_x_ on oxide) [49].

Overall, the XPS results indicate that the oxidation state of Pd species in the CS-modified catalysts is governed by the combined influence of support properties and chitosan modification. In particular, the extent of the chitosan effect appears to depend strongly on the textural characteristics of the oxide support.

### 2.2. Catalytic Properties of the Supported Pd Catalysts in Hydrogenation of 2-Propen-1-ol

**Figure 6:**
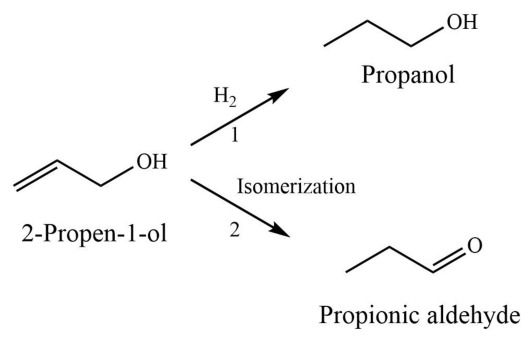
Plausible pathways of the hydrogenation of 2-propen-1-ol.

Figure 6 presents the plausible reaction pathway for the hydrogenation of 2-propen-1-ol. The hydrogenation of allyl alcohol (2-propen-1-ol) to propanol (Figure 6, reaction 1) is accompanied by a side reaction involving isomerization, resulting in propionic aldehyde (Figure 6) [6].

**Figure 7:**
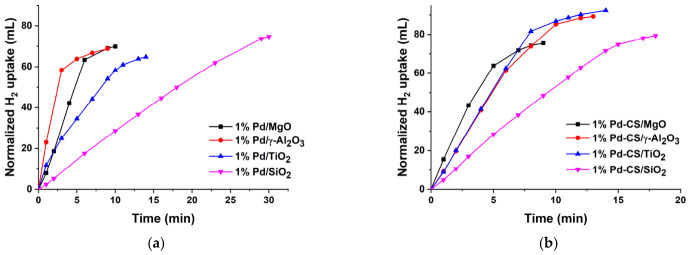
Kinetics of the hydrogen uptake over polymer-free (**a**) and chitosan-modified (**b**) Pd catalysts during the hydrogenation of 2-propen-1-ol. Reaction conditions: T—40 °C, P_H2_—1 atm, m_cat_—0.05 g, solvent C_2_H_5_OH—25 mL, substrate—0.3 mL.

The catalytic activity of the prepared Pd catalysts was investigated, and the results are presented in Figure 7. The activity of the catalysts was estimated by measuring the H_2_ uptake against time.

The semi-hydrogenation point (50 mL) was reached after 3, 5, 8 and 18 min for 1%Pd/Al_2_O_3_, 1%Pd/MgO, 1%Pd/TiO_2_, and 1%Pd/SiO_2_ catalysts, respectively. At the same time, during the initial stage of the reaction, the 1%Pd/MgO and 1%Pd/TiO_2_ catalysts exhibited relatively similar catalytic behavior, as evidenced by the hydrogen uptake kinetics (Figure 7a). The turnover frequency (TOF) values calculated from the initial reaction rates were 13100, 5150, 4940, and 1650 h^−1^ for 1%Pd/Al_2_O_3_, 1%Pd/MgO, 1%Pd/TiO_2_, and 1%Pd/SiO_2_, respectively. Thus, although the overall activity trend remained the same, the TOF analysis indicates that the intrinsic catalytic activities of the Pd/MgO and Pd/TiO_2_ systems during the initial reaction period were quite close.

The observed differences in catalytic activity are associated with the influence of the oxide support on the size, dispersion, and aggregation state of Pd nanoparticles, as confirmed by the HAADF-STEM results (Figure 4). Among the studied systems, the 1%Pd/Al_2_O_3_ catalyst exhibited the highest activity, which correlates with the formation of small Pd nanoparticles (~4 nm) uniformly distributed over the support surface. Such morphology provides a high dispersion of palladium and a larger number of accessible active sites for hydrogenation. For the 1%Pd/MgO catalyst, Pd nanoparticles were relatively uniformly distributed on the support surface with an average size of ~8 nm, although aggregates of approximately 10–30 nm were also observed. A similar situation was found for the 1%Pd/TiO_2_ catalyst, where isolated Pd nanoparticles of about 9 nm coexisted with aggregates in the range of 20–50 nm. The comparable TOF values obtained for these catalysts during the initial reaction stage suggest that both systems possess relatively similar intrinsic catalytic activity. At the same time, the somewhat higher activity of the Pd/MgO catalyst may be associated with a lower degree of Pd aggregation compared with the TiO_2_-supported system, resulting in a larger number of accessible active sites. The lowest activity was observed for the 1%Pd/SiO_2_ catalyst, which is consistent with the formation of relatively large Pd particles (~11 nm) and their strong aggregation into large clusters of approximately 100–200 nm localized on different regions of the support surface. Such extensive aggregation significantly decreases the active surface area of palladium and limits the accessibility of catalytic sites, resulting in the slowest hydrogenation rate among the investigated catalysts.

Modification of Pd catalysts with chitosan led to a partial equalization of their catalytic activity (Figure 7b). The semi-hydrogenation point for the Al_2_O_3_-, MgO-, and TiO_2_-based catalysts was reached after approximately 4–5 min, indicating similar catalytic performance after polymer incorporation, while for 1%Pd–CS(10%)/SiO_2_ it was reached after 9 min (Figure 7b). This behavior is further supported by turnover frequency (TOF) values calculated from the initial reaction rates. The overall TOF range narrowed from 1650–13100 h^−1^ for the unmodified catalysts to 3130–8840 h^−1^ after chitosan modification. At the same time, the extent of this effect depended on the nature of the oxide support. The TOF values for the chitosan-modified catalysts followed the order: 1%Pd–CS(10%)/MgO (8840 h^−1^) > 1%Pd–CS(10%)/TiO_2_ (5590 h^−1^) ≈ 1%Pd–CS(10%)/Al_2_O_3_ (5560 h^−1^) > 1%Pd–CS(10%)/SiO_2_ (3130 h^−1^). Thus, the Al_2_O_3_- and TiO_2_-supported catalysts exhibited very similar catalytic activity after chitosan modification, whereas the MgO-based system was approximately 1.6 times more active and the SiO_2_-supported catalyst showed nearly 1.8 times lower TOF value. The weaker equalization observed for the SiO_2_-supported catalyst is in line with the XPS results, where the modification-induced changes in the Pd^0^/Pd^2+^ ratio are less evident compared to the Al_2_O_3_- and TiO_2_-based systems. Although these spectroscopic changes do not provide direct evidence of their influence on catalytic activity, they indicate differences in the extent of interaction between chitosan and Pd species depending on the nature and textural characteristics of the oxide support. In the case of the MgO-supported catalyst, the higher TOF compared to the Al_2_O_3_-based system may be related to the more compact spatial distribution of Pd nanoparticles, as evidenced by HAADF-STEM analysis. Despite the generally uniform distribution of Pd and comparable nanoparticle sizes in both systems, the closer particle proximity on MgO, which also has a lower specific surface area than Al_2_O_3_, may result in a higher local density of Pd species, which could contribute to the higher catalytic activity observed for this catalyst.

Compared with the unmodified catalysts, the chitosan-modified systems exhibit noticeable changes in catalytic performance. In particular, the TOF increases from 5150 to 8840 h^−1^ for the MgO-supported catalyst, from 4940 to 5590 h^−1^ for the TiO_2_-based system, and from 1650 to 3130 h^−1^ for the SiO_2_-supported catalyst, indicating an overall enhancement of activity upon polymer incorporation. In contrast, the Al_2_O_3_-supported catalyst shows a decrease in TOF from 13100 to 5560 h^−1^ after chitosan modification. Such non-monotonic changes in catalytic activity may be related to the influence of two competing effects induced by polymer incorporation: (1) the ability of chitosan to stabilize Pd nanoparticles and improve their dispersion on the support surface, and (2) steric limitations caused by the polymer layer, which can reduce the accessibility of active Pd sites to the substrate [50,51]. For the MgO-supported catalyst, chitosan incorporation promoted a decrease in Pd particle size from 8 to 4 nm and reduced agglomeration of Pd particles (Figure 4). This structural improvement is consistent with the observed 1.7-fold enhancement in catalytic performance, suggesting a beneficial role of improved Pd dispersion in this system. In contrast, for the Al_2_O_3_-supported catalyst, the Pd particle size and distribution changed only slightly after chitosan incorporation, indicating a limited effect of the polymer on Pd dispersion. Therefore, steric hindrance associated with the polymer layer may become more relevant in this system, potentially decreasing the accessibility of active Pd centers and leading to reduced catalytic activity despite the preservation of relatively high Pd dispersion.

**Figure 8:**
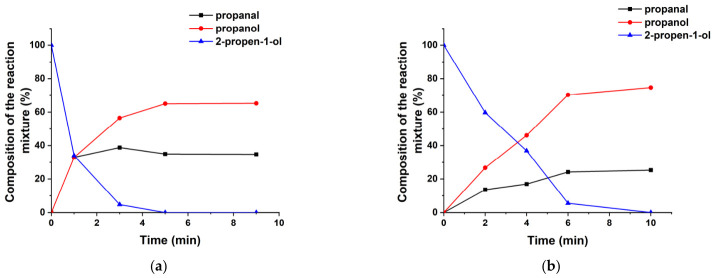

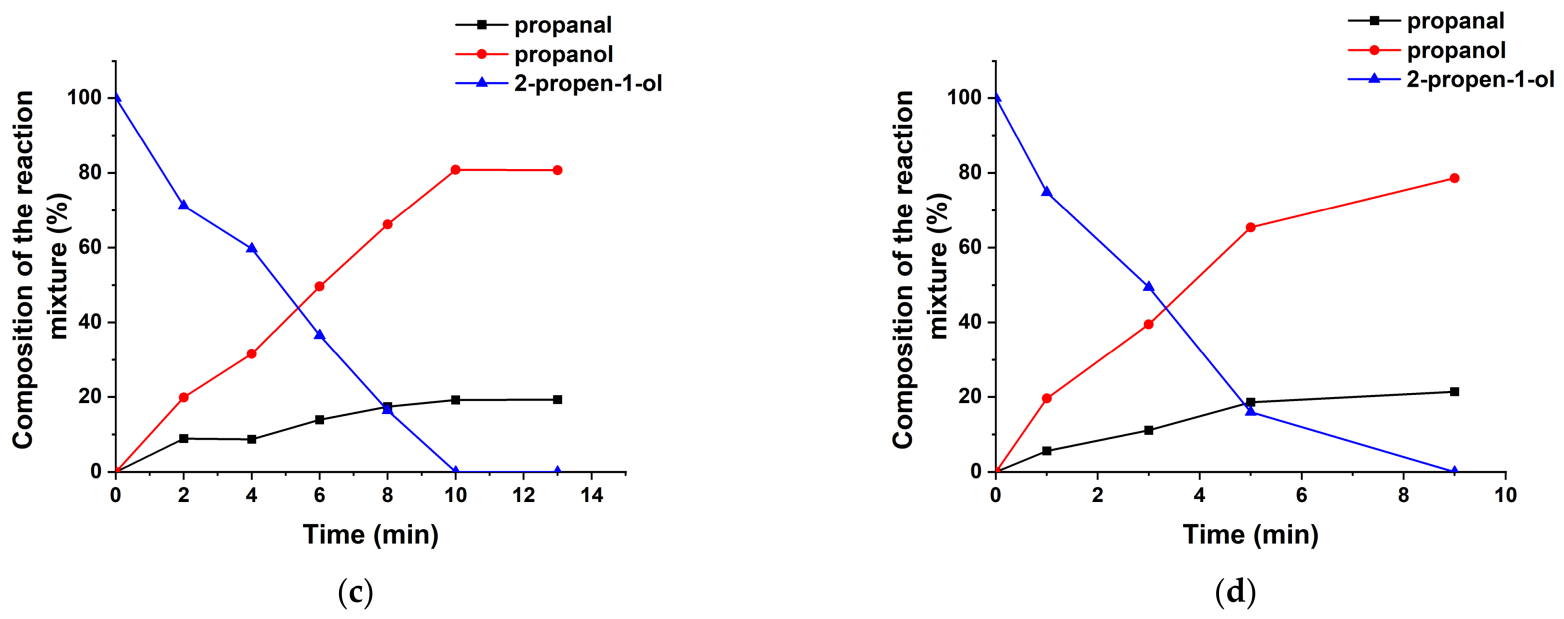
Changes in the composition of the reaction mixture during hydrogenation of 2-propen-1-ol over 1%Pd/Al_2_O_3_ (**a**), 1%Pd/MgO (**b**), 1%Pd–CS(10%)/Al_2_O_3_ (**c**), and 1%Pd–CS(10%)/MgO (**d**) catalysts. Reaction conditions: T—40 °C, P_H2_—1 atm, m_cat_—0.05 g, solvent C_2_H_5_OH—25 mL, substrate—0.25 mL.

According to the chromatographic analysis, hydrogenation of 2-propen-1-ol over the investigated catalysts resulted in the formation of both 1-propanol and propanal. In all cases, 1-propanol was the main reaction product, while propanal was formed as a product of allyl alcohol isomerization (Figure 8). In the presence of the 1%Pd/Al_2_O_3_ catalyst, the maximum propanol yield reached 65% after 5 min at complete conversion of 2-propen-1-ol (Figure 8a). For the 1%Pd/MgO catalyst, the maximum propanol yield was 75% after 10 min at 100% substrate conversion (Figure 8b). Over the 1%Pd–CS(10%)/Al_2_O_3_ catalyst, the maximum propanol yield reached 81% after 10 min at complete substrate conversion (Figure 8c). In the case of the 1%Pd–CS(10%)/MgO catalyst, the propanol yield reached 79% after 9 min at 100% conversion of 2-propen-1-ol (Figure 8d). Thus, modification of Pd catalysts with chitosan generally increased the yield of propanol compared with the corresponding unmodified catalysts. A similar trend was also observed for the remaining investigated catalysts (Figure S1).

The chromatographic analysis data over the catalysts prepared are summarized in Table 3. The selectivity toward propanol was strongly dependent on the nature of the support. For Pd/Al_2_O_3_ and Pd/TiO_2_ catalysts, the propanol selectivity was 65 and 67%, respectively. In contrast, Pd/MgO and Pd/SiO_2_ exhibited higher selectivity values of 75 and 72%, respectively.

The lower selectivity to propanol observed for Al_2_O_3_- and TiO_2_-supported catalysts can be attributed to the presence of Lewis acidic surface sites of the supports. In this context, Zsolnai et al. reported that alumina-supported Pd exhibits activity in allyl alcohol isomerization, where Lewis acidic sites participate in alcohol activation toward oxidative dehydrogenation by the metal, followed by hydride transfer [52].

After modification with chitosan, the selectivity of the Pd catalysts toward propanol increased to 76–81%. The enhancement in selectivity was most pronounced for the Al_2_O_3_-supported catalyst (81%) and least significant for the SiO_2_-based system (76%). The improvement in selectivity to propanol after chitosan modification correlates with changes in the Pd^0^/Pd^2+^ ratio, suggesting an alteration in the local environment of Pd species upon interaction with the polymer. These changes are more pronounced for the Al_2_O_3_- and TiO_2_-supported catalysts compared with the SiO_2_-based system. This observation is consistent with literature reports demonstrating that modification of the coordination environment of Pd nanoparticles can influence catalytic activity and selectivity [15]. In addition, it has been reported [52] that Lewis acidic sites of Al_2_O_3_ can interact with Pd and participate in the activation of allyl alcohol, promoting the isomerization pathway. In the present work, HAADF-STEM and XPS results for the Al_2_O_3_-based catalyst indicate a noticeable change in the Pd environment after chitosan modification. This may affect the interaction between Pd sites and the oxide support surface, thereby contributing to the observed changes in product distribution and selectivity.

Overall, the results demonstrate that in unmodified systems catalytic activity and selectivity are mainly governed by Pd dispersion and metal–support interactions. Chitosan modification restructures the local catalytic environment of Pd, enhances hydrogenation selectivity, and reduces the dependence of catalytic performance on the nature of the oxide support.

**Figure 9:**
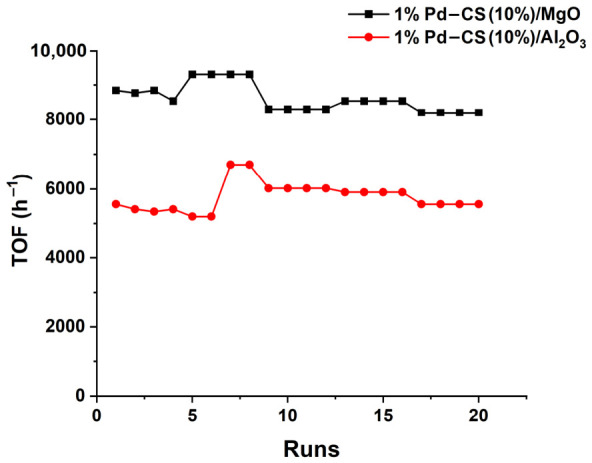
Reuse of 1%Pd–CS/MgO and 1%Pd–CS/Al_2_O_3_ catalysts in hydrogenation of 2-propen-l-ol.

The stability of the 1%Pd–CS(10%)/Al_2_O_3_ and 1%Pd–CS(10%)/MgO catalysts was evaluated in the hydrogenation of successive portions of 2-propen-1-ol using the same catalyst sample (Figure 9). Both catalysts demonstrated generally stable performance over 20 catalytic cycles without significant decrease in catalytic activity. The Pd content in the catalysts after 20 catalytic cycles remained within 1.0–1.1 wt.% (Table S1), indicating no significant Pd loss during repeated catalytic cycles. Similar behavior was previously reported for Pd/chitosan catalytic systems, in which only negligible Pd leaching was detected during catalytic operation [42]. In addition, scanning electron microscopy (SEM) images of the catalysts did not reveal noticeable changes in their morphology after 20 catalytic cycles, confirming their structural stability (Figure S2).

When compared with other reported palladium-based catalysts for the hydrogenation of 2-propen-1-ol, the 1%Pd–CS(10%)/MgO and 1%Pd–CS(10%)/Al_2_O_3_ catalysts exhibit comparable substrate conversion and selectivity toward propanol (Table 4). The turnover frequency (TOF) for 1%Pd–CS(10%)/MgO is 8840 h^−1^, which is close to that of other hydrogenation catalysts, such as Pd(0)–EGCG0.2–CF (9703 h^−1^) [12].

## 3. Materials and Methods

### 3.1. Materials

Chitosan (CS, Mw 250,000), magnesium oxide (MgO, pure grade), alumina (Al_2_O_3_, pure grade), titanium dioxide (TiO_2_, anatase, 99.7%), palladium (II) chloride (PdCl_2_, Pd content 59–60%), potassium chloride (KCl, pure grade), ammonium hydroxide (NH_4_OH), aluminum chloride (AlCl_3_·6H_2_O, pure grade), 2-propen-1-ol were purchased from Sigma-Aldrich, St. Louis, MO, USA. Sodium borohydride (NaBH_4_, 95%) were acquired from AppliChem, Darmstadt, Germany. Ethanol (reagent grade) was obtained from Talgar Alcohol LLP (Talgar, Kazakhstan).

### 3.2. Synthesis of Alumina

Alumina (γ-Al_2_O_3_) was synthesized by precipitation in ethanol according to the method described in [32]. Ammonium hydroxide was used as the precipitating agent. For this purpose, 200 mL of a 4 M aqueous NH_4_OH solution was added dropwise to a freshly prepared solution of aluminum chloride (94.6 g of AlCl_3_·6H_2_O dissolved in 300 mL of ethanol) under vigorous stirring. The resulting white gel-like precipitate was filtered, washed with ethanol, and dried at 100 °C for 2 h. The dried sample was calcined at 1029 °C for 2 h.

### 3.3. Synthesis of Pd Catalysts

Polymer-modified palladium catalysts containing 10% chitosan were prepared as follows [14]. A 20 mL portion of a 3.3 × 10^−2^ M chitosan solution (0.11 g dissolved in 20 mL of 1% hydrochloric acid) was introduced gradually into a mixture of 1 g of the support material (MgO, Al_2_O_3_, TiO_2_ or SiO_2_) in 15 mL of water at ambient temperature. After addition of the polymer solution, the pH of the system was adjusted to 7.5 under continuous stirring. The resulting CS/oxide support composite was then stirred for an additional 2 h. Subsequently, the resulting flocculent precipitate was allowed to settle, and the clear mother liquor was separated for viscosity measurements.

The polymer content in the composites was estimated from the change in chitosan concentration in the mother liquor before and after deposition using a calibration curve describing the dependence of viscosity on chitosan concentration. Viscosity measurements were carried out at 20 °C using an Ubbelohde viscometer and a thermostatic bath (KRIO-VIS-E-01, Termex, Tomsk, Russia), and flow times were averaged over at least three measurements. Calibration curves were obtained from chitosan solutions of known concentration prepared under identical conditions (same polymer batch, solvent). The chitosan concentration after deposition was determined from the calibration curve, and the amount of adsorbed polymer was calculated from the difference between initial and final concentrations. The method provides a semi-quantitative estimation of polymer loading.

Subsequently, 5 mL of a 1.9 × 10^−2^ M potassium tetrachloropalladate(II) (K_2_PdCl_4_) solution and 3 mL of a freshly prepared 0.13 M solution of sodium borohydride (0.015 g in 3 mL of water) were gradually introduced into the resulting chitosan-containing support system under continuous stirring. The hybrid system was then stirred for an additional 3 h. The amount and concentration of the palladium precursor (5 mL of 1.9 × 10^−2^ M potassium tetrachloropalladate(II) solution) were selected based on calculations to obtain catalysts containing 1 wt.% palladium. The Pd/MgO, Pd/TiO_2_, Pd/Al_2_O_3_ and Pd/SiO_2_ samples without polymer were synthesized following the same procedure, but without the addition of the polymer component.

### 3.4. Characterisation of the Catalysts by Physicochemical Methods

Powder X-ray diffraction (XRD) patterns were obtained with a DRON-4-0.7 X-ray diffractometer (Bourevestnik, Saint Petersburg, Russia) using cobalt-monochromatized Co Kα radiation (λ = 0.179 nm). Scanning electron microscopy (SEM) images were recorded using a JSM-6610 LV scanning electron microscope (JEOL Ltd., Tokyo, Japan) operating at an accelerating voltage of 15–20 kV. Elemental composition was determined with a JSM-6610LV (JEOL, Tokyo, Japan) SEM equipped with an energy-dispersive X-ray (EDX) detector system [53]. An elemental analyzer Vario Micro cube (Elementar Analysensysteme GmbH, Langenselbold, Germany) was used for the organic microanalysis of the samples. The contents of carbon, hydrogen, and nitrogen (CHN) were determined by high-temperature combustion at 1150 °C under a stream of pure oxygen. The resulting combustion products were then reduced and separated on a chromatographic column equipped with a thermal conductivity detector. Thermogravimetric analysis (TGA) of the catalyst was carried out in a nitrogen atmosphere (50 mL/min) at temperature range of 30–600 °C using a STA 449F5 analyzer (Netzsch, Selb, Germany) at a heating rate of 20 °C per minute. X-ray photoelectron spectroscopy (XPS) measurements of the samples were performed using an ESCALAB 250Xi X-ray and Ultraviolet photoelectron spectrometer (Thermo Fisher Scientific, Waltham, MA, USA) equipped with Al Kα radiation (photon energy 1486.6 eV). Spectra were collected in constant pass energy mode, with 50 eV for survey scans and 20 eV for core-level spectra, using an XPS analysis area of 650 μm. The overall energy resolution of the measurements was approximately 0.3 eV. All analyses were conducted at room temperature under ultrahigh vacuum conditions of about 1 × 10^−9^ mbar. Sample charging was compensated using an ion-electron neutralization system. High-angle annular dark-field scanning transmission electron microscopy (HAADF-STEM) images were acquired using a Zeiss Libra 200FE transmission electron microscope (Carl Zeiss, Oberkochen, Germany) operating at an accelerating voltage of 100 kV [53].

### 3.5. Methodology of Hydrogenation

The hydrogenation of the 2-propen-1-ol was carried out in a thermostated glass reactor according to the procedure described in Ref. [14]. The hydrogenation of unsaturated compounds was carried out with H_2_ from a gas storage burette connected to the reactor in an ethanol medium (25 mL) at atmospheric hydrogen pressure and a temperature of 40 °C, under intensive stirring (600–700 oscillations per minute) [14]. Before hydrogenation, the catalyst (0.05 g) was reduced with hydrogen (1 atm) in a reactor at 40 °C for 30 min under conditions of intensive stirring. After the hydrogen treatment, a substrate (0.3 mL of 2-propen-1-ol) was added to the reactor. Catalytic activity was evaluated from the initial hydrogen uptake rates, and turnover frequency (TOF) values were calculated on the basis of the hydrogen consumption rate during the initial stage of the reaction. Hydrogen uptake was quantified by monitoring the H_2_ volume in a gas burette connected to the reactor [53].

The products of hydrogenation were investigated by gas chromatography using a Chromos GC-1000 instrument (Chromos, Dzerzhinsk, Russia) equipped with a flame ionization detector. Separation was carried out on a BP21 (FFAP) capillary column with a polar stationary phase (PEG modified with nitroterephthalate), 50 m in length and 0.32 mm internal diameter. Catalyst selectivity was defined as the ratio of the desired product to the total amount of all reaction products at a given conversion.

Catalyst reusability was assessed over successive cycles (0.3 mL, 2.23 mmol) using the same catalyst sample (50 mg) at 40 °C under atmospheric hydrogen pressure [53], based on TOF values derived from initial hydrogenation rates.

## 4. Conclusions

A series of palladium catalysts supported on MgO, SiO_2_, TiO_2_, and Al_2_O_3_, both chitosan-modified and polymer-free, were successfully prepared by the precipitation method. EDX elemental analysis confirmed that all catalysts contain a comparable palladium loading in the range of 0.9–1.3 wt.%. For the polymer-modified systems, viscosity measurements, TGA, and CHN analysis consistently indicated a similar chitosan content of approximately 10 wt.%, demonstrating effective and reproducible incorporation of the polymer across all supports.

HAADF-STEM analysis revealed a strong dependence of Pd nanoparticle dispersion on the nature of the oxide support. Among the polymer-free catalysts, Al_2_O_3_ provided the most uniform distribution of small Pd nanoparticles with an average size of ~4 nm and no pronounced aggregation. In contrast, SiO_2_, TiO_2_, and MgO exhibited larger Pd particles (8–11 nm) together with partial aggregation, with the most severe agglomeration observed for the SiO_2_-supported catalyst. This behavior is attributed to weak interactions between Pd precursor species and the strongly acidic silica surface, which limits effective stabilization during deposition and promotes particle growth during reduction, whereas Al_2_O_3_ provides stronger anchoring of Pd species and suppresses aggregation. MgO shows intermediate dispersion behavior, where partial aggregation is observed despite favorable surface chemistry, likely due to limited surface area. Chitosan modification significantly improved Pd dispersion, reducing particle size to ~3–4 nm and suppressing agglomeration, particularly for MgO-supported catalysts. This effect can be attributed to the ability of chitosan functional groups (–NH_2_ and –OH) to coordinate Pd precursor species, promoting their stabilization within the polymer matrix and limiting uncontrolled nucleation and growth during reduction.

XPS results revealed that both the oxide support and chitosan modification strongly influence the electronic state of palladium. In the polymer-free catalysts, Pd/Al_2_O_3_ and Pd/SiO_2_ exhibit comparable fractions of Pd^2+^ species, whereas Pd/TiO_2_ shows a lower Pd^2+^ content together with a slight negative shift in the Pd 3d binding energy, indicating differences in metal–support electronic interactions. After chitosan incorporation, an overall increase in the fraction of oxidized Pd species is observed for all catalysts, indicating a general modification of the Pd electronic environment. However, the magnitude of this effect depends on the nature of the support, with more pronounced changes observed for TiO_2_- and Al_2_O_3_-supported catalysts compared with SiO_2_-based systems.

The catalytic performance of Pd-based systems in the low-temperature hydrogenation of 2-propen-1-ol is strongly governed by the nature of the oxide support, which determines Pd dispersion and aggregation, thereby controlling catalytic activity, while also influencing selectivity through metal–support interactions and the acid–base properties of the support surface. Chitosan modification significantly alters the structure–activity relationship of the investigated Pd catalysts. In contrast to the unmodified systems, where catalytic activity is primarily governed by Pd dispersion and aggregation, polymer incorporation introduces additional effects associated with changes in nanoparticle stabilization and accessibility of active sites. As a result, catalytic activity becomes less strictly dependent on the initial support characteristics, although the extent of this effect remains support-dependent, with MgO-, TiO_2_-, and SiO_2_-supported catalysts showing an increase in activity, while a decrease is observed for the Al_2_O_3_-based system. In addition to activity changes, chitosan modification leads to a pronounced improvement in selectivity toward propanol formation for all investigated catalysts. This effect is more significant for Al_2_O_3_- and TiO_2_-supported systems, while SiO_2_-based catalysts show a comparatively weaker response. The increase in selectivity is attributed to the modification of the Pd environment induced by the polymer, which alters reaction pathways and suppresses competing side reactions associated with the intrinsic properties of the oxide supports. Stability tests performed for Pd–CS(10%)/MgO and Pd–CS(10%)/Al_2_O_3_ catalysts demonstrated stable performance over 20 catalytic cycles without significant loss in activity or appreciable Pd leaching.

Overall, chitosan modification of supported Pd catalysts provides an effective strategy to tune both the catalytic activity and selectivity of palladium-based systems, while the oxide support, particularly its textural characteristics, modulates the extent of these effects at a given polymer loading. The combined influence of nanoparticle size, support properties, and Pd–chitosan coordination determines the catalytic behavior, highlighting the potential of these hybrid systems for selective hydrogenation processes, including the valorization of biomass-derived platform molecules into valuable chemical products.

## Figures and Tables

**Table 1:** The EDX elemental analysis of the studied samples.

Sample	Mass, %						
	O	Mg	Al	Si	Ti	Na	Pd
Pd/MgO	39.1	60.0	–	–	–	–	0.9
1% Pd-CS(10%)/MgO	49.2	49.4	–	–	–	0.3	1.1
Pd/γ-Al_2_O_3_	47.9	–	49.9	–	–	0.9	1.3
1% Pd-CS(10%)/Al_2_O_3_	46.6	–	50.5	0.8	0.9	–	1.2
Pd/SiO_2_	53.8	0.6	0.2	44.1	–	0.3	1.0
1% Pd-CS(10%)/SiO_2_	54.0	1.0	0.4	43.4	–	–	1.2
Pd/TiO_2_	42.8	–	0.3	–	55.6	–	1.3
1% Pd-CS(10%)/TiO_2_	44.3	–	–	0.6	53.8	–	1.3

**Table 2:** Elemental analysis for chitosan and CS-modified palladium catalysts.

Sample	Content, %		
	C	H	N
Chitosan	42.1	7.5	7.4
1%Pd-CS(10%)/MgO	5.5	1.6	0.2
1%Pd-CS(10%)/Al_2_O_3_	5.6	0.9	0.6
1%Pd-CS(10%)/TiO_2_	4.6	0.8	0.5
1%Pd-Xиt(10%)/SiO_2_	4.5	1.1	0.4

**Table 3:** Catalytic properties of Pd/support and Pd-CS/support catalysts in the hydrogenation of 2-propen-1-ol.

Catalyst	Selectivity, %		Conversion, %
	Propanal	Propanol	
1%Pd/Al_2_O_3_	35	65	100
1%Pd-CS(10%)/Al_2_O_3_	19	81	100
1%Pd/MgO	25	75	100
1%Pd-CS(10%)/MgO	21	79	100
1%Pd/TiO_2_	33	67	100
1%Pd-CS(10%)/TiO_2_	22	78	100
1%Pd/SiO_2_	28	72	100
1%Pd-CS(10%)/SiO_2_	24	76	100

**Table 4:** Comparative study of 1%Pd–CS(10%)/MgO and 1%Pd–CS(10%)/Al_2_O_3_ with other palladium catalysts for the hydrogenation of 2-propen-1-ol.

Catalyst	T, °C	Pressure, MPa	TOF, h^−1^	Propanol Selectivity, %	Conversion, %	Ref.
Pd CaLig NPs	20	0.1	-	74.0	100	[6]
Commercial Pd/C	20	0.1	-	71.0	100	[6]
C16NH_2_ Pd	r.t.	0.1	114	73.0	100	[20]
Pd/Zr_6_O_4_(OH)_6_(C_8_H_4_O_4_)_6_(H_2_O)_4_	40	0.1	-	78.0	100	[22]
Pd(0)–EGCG0.2–CF	30	0.5	9703	89.0	99.8	[12]
1%Pd-CS(10%)/MgO	40	0.1	8840	78.6	100	This study
1%Pd-CS(10%)/Al_2_O_3_	40	0.1	5560	80.7	100	This study

## Data Availability

The data that support the findings of this study are available from the corresponding author upon reasonable request.

## Supplementary Materials

The following supporting information can be downloaded at: https://www.mdpi.com/article/10.3390/molecules31122028/s1, Figure S1: Changes in the composition of the reaction mixture during the hydrogenation of 2-propen-1-ol in the presence of 1%Pd/TiO_2_ (a), 1%Pd/SiO_2_ (b), 1%Pd-CS(10)/TiO_2_ (c) and 1%Pd-CS(10%)/SiO_2_ (d); Figure S2: SEM images of the initial 1%Pd-CS(10%)/Al_2_O_3_ (a), 1%Pd-CS(10%)/MgO (c), and the same catalysts after 20 cycles of 2-propen-1-ol hydrogenation: 1%Pd-CS(10%)/Al_2_O_3_ (b) and 1%Pd-CS(10%)/MgO (d); Table S1: The EDX elemental analysis of the spent samples.
