# Supplementary material for: Effect of Chitosan Modification and Support Type on the Catalytic Properties of Supported Palladium Catalysts in Hydrogenation of 2-Propen-1-ol
Source: Molecules. 2026 Jun 10;31(12):2028. doi: 10.3390/molecules31122028 (PMC13306000; doi:10.3390/molecules31122028)
Supplement: Supplementary file 1 [file molecules-31-02028-s001.zip › molecules-4291127-supplementary.pdf]

Supplementary Information for:

Effect of Chitosan Modification and Support Type on the Catalytic Properties of Supported Palladium Catalysts in Hydrogenation of 2-Propen-1-ol

Akzhol Naizabayev, Eldar Talgatov, Assemgul Auyezkhanova *, Arlan Abilmagzhanov, Sandugash Akhmetova, Alima Kenzheyeva and Raiymbek Yersaiyn

D.V. Sokolsky Institute of Fuel, Catalysis, and Electrochemistry, Kunaev Str. 142, Almaty 050010, Kazakhstan

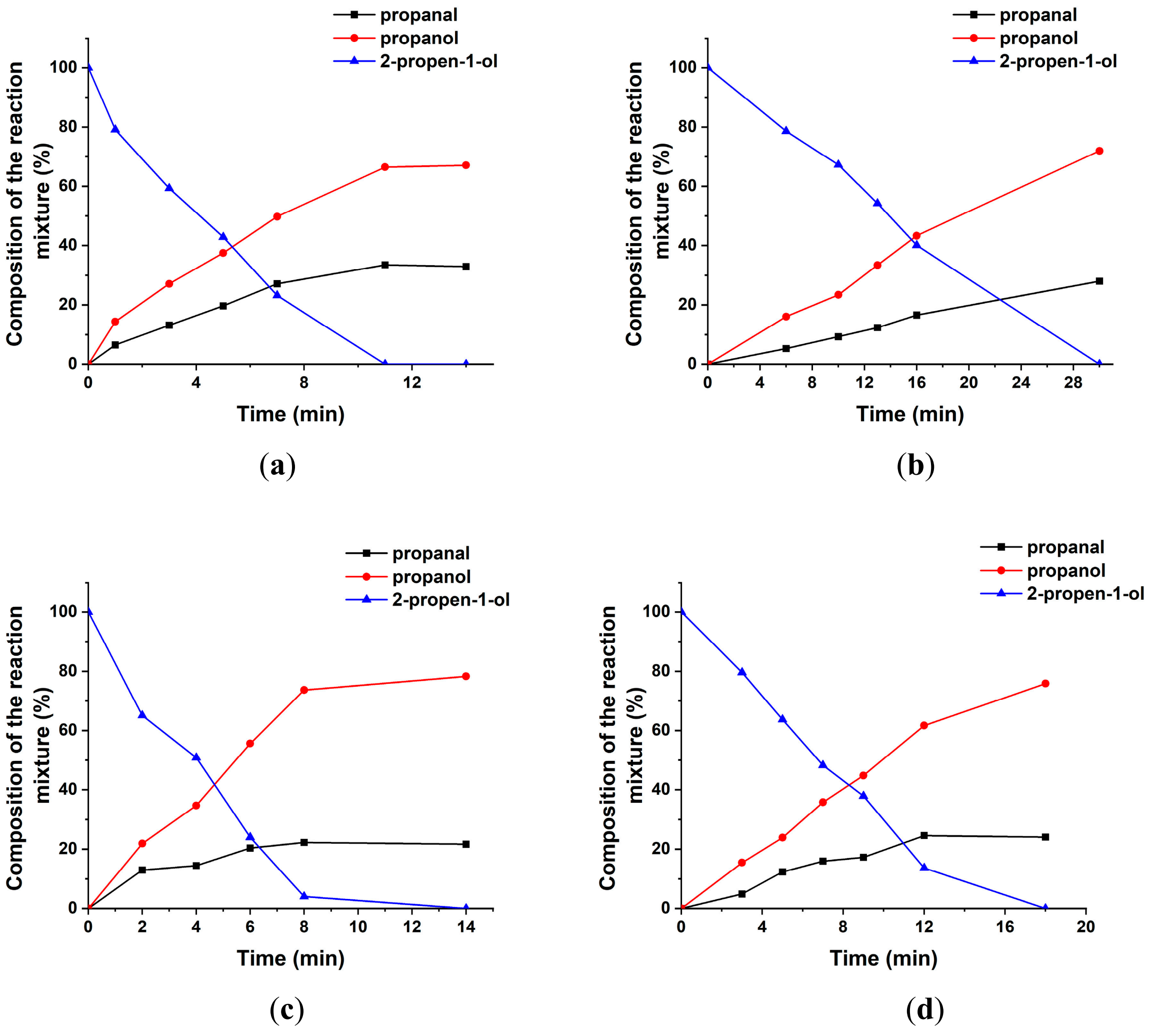

Figure. S1. Changes in the composition of the reaction mixture during the hydrogenation of 2-propen-1-ol in the presence of 1%Pd/TiO_2 (**a**), 1%Pd/SiO_2 (**b**), 1%Pd-CS(10)/TiO_2 (**c**) and 1%Pd-CS(10%)/SiO_2 (**d**). Reaction conditions: T—40 °C, P_{H2}—1 atm, m_{cat}—0.05 g, solvent C_2H_5OH—25 mL, substrate—0.25 mL.

Table S1. The EDX elemental analysis of the spent samples.

**Sample**	**Mass, %**						
	**O**	**Mg**	**Al**	**Si**	**Ti**	**Na**	**Pd**
1% Pd-CS(10%)/MgO (after 20 cycles)	49.3	48.2	0.5	1.0	–	–	1.0
1% Pd-CS(10%)/Al_2O_3 (after 20 cycles)	49.5	–	47.9	0.7	0.8	–	1.1

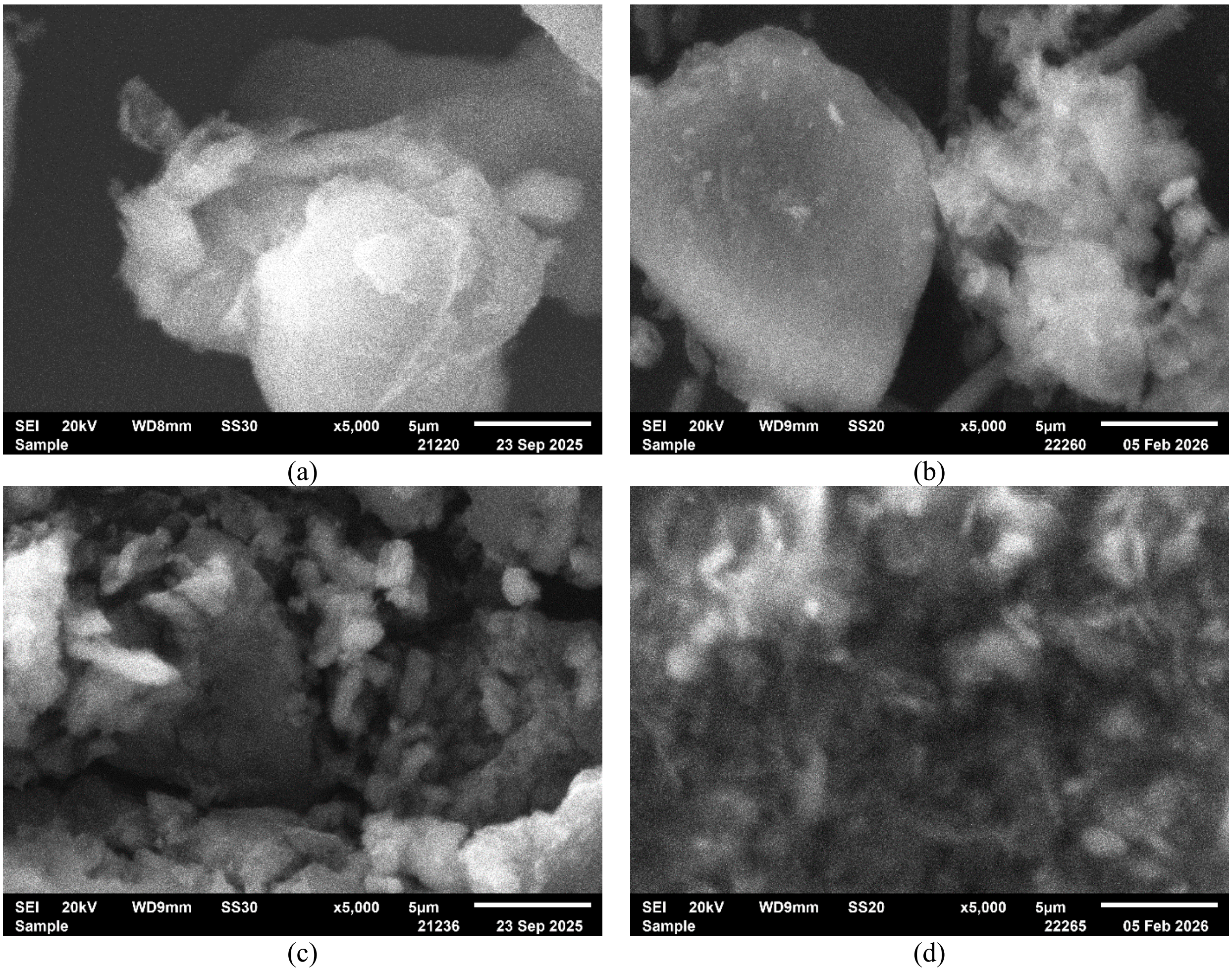

Figure S2. SEM images of the initial 1%Pd-CS(10%)/Al_2O_3 (a), 1%Pd-CS(10%)/MgO (c), and the same catalysts after 20 cycles of 2-propen-1-ol hydrogenation: 1%Pd-CS(10%)/Al_2O_3 (b) and 1%Pd-CS(10%)/MgO (d).
